# ZIF-8-derived hybrid nanocomposite platform with magnetic hematite nanoparticles as enhanced anode materials for lithium storage†

**DOI:** 10.1039/d5ra01206f

**Published:** 2025-04-22

**Authors:** Do Thao Anh, Nguyen Bao Tran, Nguyen La Ngoc Tran, Tran Huu Huy, Tran Thi Kim Chi, Tran Thi Huong Giang, Van Man Tran, Nguyet N. T. Pham, Tuan Loi Nguyen, Nhu Hoa Thi Tran

**Affiliations:** a Center for Innovative Materials and Architectures (INOMAR) Ho Chi Minh City 700000 Vietnam; b Vietnam National University Ho Chi Minh City 700000 Vietnam ttnhoa@hcmus.edu.vn; c Faculty of Materials Science and Technology, University of Science Ho Chi Minh City 700000 Vietnam; d Quy Nhon College of Engineering and Technology Quy Nhon 590000 Vietnam; e Institute of Materials Science, Vietnam Academy of Science and Technology Hanoi Vietnam; f Applied Physical Chemistry Laboratory (APCLAB), University of Science Ho Chi Minh City 700000 Vietnam; g Department of Physical Chemistry, Faculty of Chemistry, University of Science Ho Chi Minh City 700000 Vietnam; h Institute of Fundamental and Applied Sciences, Duy Tan University Ho Chi Minh City 70000 Vietnam nguyentuanloi@duytan.edu.vn; i Faculty of Environmental and Chemical Engineering, Duy Tan University Da Nang City 50000 Vietnam

## Abstract

Herein, α-Fe_2_O_3_–ZnO/C (FZC) nanocomposite samples were synthesized *via* a chemical co-precipitation method, followed by a one-step heat-treatment at different temperatures to serve as anode materials. The advantages of FZC include a high specific surface area, a porous structure that facilitates rapid ion/electron transport, and additional active sites for lithium ions, leading to excellent electrical conductivity and superior electrochemical performance. The FZC4 material demonstrated a high charge/discharge capacity of 561.2/587.8 mA h g^−1^ after 80 cycles at a current density of 0.1 A g^−1^, with low impedance and a coulombic efficiency (CE) of 95.4%. The outstanding electrochemical performance of the FZC nanocomposites can be attributed to the synergistic effect between the hematite (α-Fe_2_O_3_) nanoparticles and ZIF-8-derived platform framework, which significantly enhanced the lithium storage capacity of the anode. Our work provides an additional contribution to the field of nanomaterial research, expanding the potential for developing efficient and sustainable energy storage solutions in the future.

## Abstract 2

α-Fe_2_O_3_–ZnO/C nanocomposite as a high-performance anode for LIBs.

## Graphical abstract

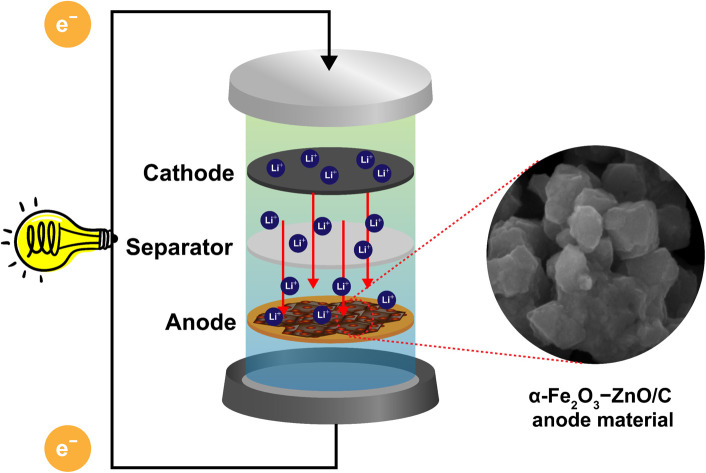

## Introduction

Currently, fossil fuels remain the primary and most important energy source, despite their increasing depletion and significant negative environmental impacts. Simultaneously, renewable energy sources, such as solar, wind, and geothermal energy, are being extensively harnessed, offering promising developments. However, the development of efficient storage systems to store energy from these sources remains a major challenge for the scientific community. Under the pressure of rising energy consumption demands, rechargeable lithium-ion batteries (LIBs) have become a focal point of research owing to their outstanding advantages, including high energy density, low charge loss, lack of memory effect, long lifespan, and fast charging speed.^1,2^ However, current commercial LIBs still face limitations in terms of specific capacity, durability, production costs, and safety. As a result, numerous studies have focused on developing new materials to enhance the performance of electrode structures, electrolytes, and separators to address these shortcomings. Among the components of LIBs, electrodes play the most critical role as they are responsible for storing charges through the reversible conversion of chemical energy into electrical energy. In particular, the anode has garnered significant attention owing to its low operating voltage and potential for much higher capacity improvements compared with the cathode. Currently, commercial LIBs use graphite-based anodes, but they offer a low theoretical capacity (372 mA h g^−1^), creating a barrier to improving the overall performance of these batteries.^3^

Transition metal oxides (TMOs) have been identified as potential alternatives to graphite for use as anode materials in LIBs owing to their superior advantages, such as exceptionally high specific capacities. Moreover, the reversible reaction of lithium ions with metal oxides helps in minimizing the formation of lithium-metal alloys,^2^ as described in eqn (1).

1 MO + *x*Li^+^ + *x*e^−^ ↔ LiO_2_ + M (M = Fe, Co, Ni…)

The forward reaction is thermodynamically favorable, enabling electron transfer for each metal atom and resulting in a theoretically high capacity for lithium storage. However, the reverse reaction is thermodynamically unfavorable. It is believed that the formation of metal nanoparticles (M) during the reaction facilitates the reverse process. This indicates that the reversibility of the reaction is well-maintained at the nanoscale.^2^ Among the various transition metal oxides, hematite nanoparticles (α-Fe_2_O_3_ NPs) have garnered significant attention owing to their high theoretical capacity of 1060 mA h g^−1^, abundance, environmental friendliness, and low synthesis cost.^4–6^ Therefore, α-Fe_2_O_3_ NPs have emerged as promising candidates for the fabrication of LIB anodes. However, their low electrical conductivity, significant volume changes (∼200%), and severe capacity loss during prolonged cycling pose substantial challenges to their application in secondary batteries.^7^ To address this issue, α-Fe_2_O_3_ NPs with a highly porous structure can be constructed to provide a buffer space for the volume changes of α-Fe_2_O_3_ NPs.

Metal–organic frameworks (MOFs) are formed through the combination of metal ions (or metal clusters) with electron-donating organic ligands and offer advantages such as high surface area, adjustable pore sizes, and controllable structure and morphology.^8^ Zeolitic imidazolate framework-8 (ZIF-8), a subclass of MOFs, consists of tetrahedral Zn metal ions linked by imidazolate ligands, thus inheriting the benefits of both MOFs and zeolites.^9^ Since its discovery, ZIF-8 has attracted significant attention due to its large surface area, high porosity, ease of size and morphology control, biocompatibility, low toxicity, and thermal and chemical stability.^9–12^ However, the electrochemical performance of ZIF-8 as an anode material for LIBs is hindered by its poor conductivity and irreversible Li^+^ storage. Further negative effects include the breakdown of the reduced electrolyte and the development of a solid-electrolyte interface (SEI) layer.^13,14^ These factors have restricted its usefulness in energy storage application.

There have been many attempts to improve this drawback of ZIF-8 or use it as a sacrificial precursor or porous carbon framework anchoring other active materials. Xiong Wen David Lou *et al.*^15^ reported the use of an MOF-assisted strategy to synthesize Fe_2_O_3_ nanotubes encapsulated in a Co_3_O_4_ host *via* thermal treatment. This hierarchical nanostructure enhanced the lithium storage properties, offering excellent cycling stability and rate capability for LIB anodes. In the report by Yan *et al.*,^16^ a phosphorus-carbon composite derived from ZIF-8 improved and stabilized the long-term cycling performance for batteries by providing a buffer space for volume expansion. Zhang *et al.*^17^ synthesized ZnO@carbon with a porous nano-cage structure derived from ZIF-8, which not only buffered the volume expansion of ZnO nanoparticles but also prevented their aggregation. This enhancement improved the electrochemical reaction kinetics and mechanical stability of the electrode material. Recently, Pan *et al.*^18^ developed a 3D self-standing carbon tube network (3D-CT) interconnected with α-Fe_2_O_3_ NPs encapsulated within the CT walls (3D-CT@ Fe_2_O_3_-NPs@C). This structure, featuring open channels, not only provided abundant anchoring sites for α-Fe_2_O_3_ NPs and accommodated the strain from α-Fe_2_O_3_ volume changes but also facilitated rapid ion and electron transport. Meanwhile, Huang *et al.*^4^ reported the preparation of a core–shell Fe_2_O_3_@N-doped carbon electrode by coating ZIF-8 on α-Fe_2_O_3_ NPs, followed by carbonization. This structure enabled long-term cycling stability for batteries. These efforts highlight the extensive endeavors by scientists to address the challenges associated with α-Fe_2_O_3_ and ZIF-8, paving the way for breakthroughs in anode material research for LIBs.

In this study, we introduce a straightforward approach to synthesize α-Fe_2_O_3_–ZnO/C (FZC) nanocomposites *via* the co-precipitation method, followed by high-temperature heat treatment for use as an anode material in LIBs. This hybrid composite offers a promising solution for LIB anodes due to its high reversibility, stable cycling performance, and excellent rate capability, stemming from the synergistic properties of its components.

## Experimental

### Materials

The chemicals used in material synthesis included iron(iii) chloride hexahydrate (FeCl_3_·6H_2_O, 99%), iron(ii) chloride tetrahydrate (FeCl_2_·4H_2_O, 99%), zinc nitrate hexahydrate (Zn(NO_3_)_2_·6H_2_O, 98%), 2-methylimidazole (C_6_H_4_N_2_, 99%), polyvinylpyrrolidone (PVP, average *M*_w_ ∼55 000), poly(vinylidene fluoride) (PVDF, average *M*_w_ ∼534 000) and *N*-methyl pyrrolidone (NMP), which were acquired from Sigma-Aldrich. All materials were of analytical grade and utilized as supplied, without any additional purification.

### Synthesis of α-Fe_2_O_3_–ZnO/C hybrid

**Scheme 1:**
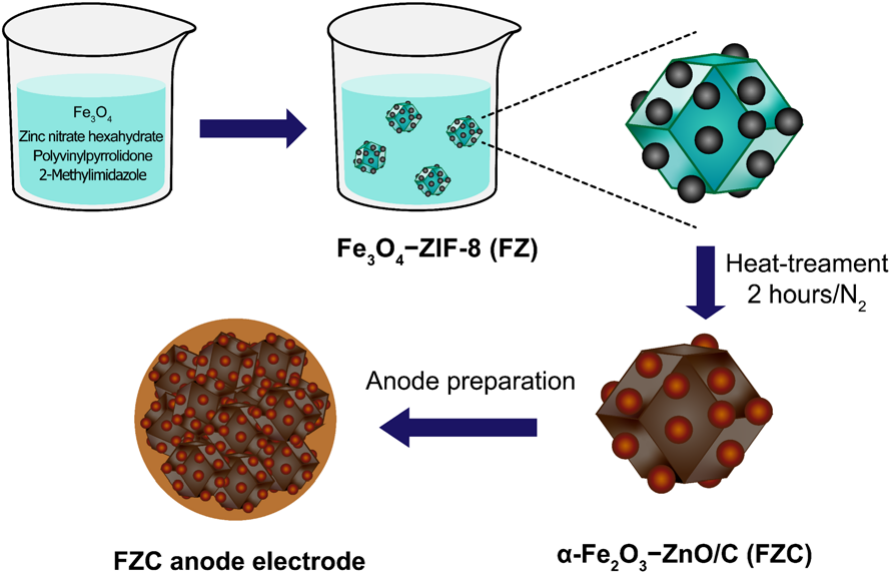
Schematic of the synthesis process for the anode electrode based on FZC nanocomposite.

The ZIF-8 and Fe_3_O_4_ NPs were prepared following our previously reported procedures.^19,20^ The Fe_3_O_4_-ZIF-8 (FZ) sample was first synthesized by a co-precipitate synthesis method. A mixture of Fe_3_O_4_ (400 mg and 500 mg, respectively), zinc nitrate hexahydrate (713.6 mg), 2-methylimidazole (394.4 mg), and polyvinylpyrrolidone (550 mg) was dissolved in 80 mL of methanol and stirred for 24 h. Then, the resulting mixture was centrifuged and washed several times with methanol to remove impurities, followed by drying under vacuum at 80 °C. Subsequently, the resulting material was heat-treated under nitrogen for 2.5 h to produce the α-Fe_2_O_3_–ZnO/C (FZC) nanocomposite material. The resulting materials were designated as FZ4 and FZ5, corresponding to 400 mg and 500 mg of Fe_3_O_4_, respectively. In the case of the heat-treated samples, FZC4 and FZC5 were obtained at 700 °C, while FZC4-300 and FZC4-500 were prepared at 300 °C and 500 °C, respectively, for comparison. The synthesis procedure is illustrated in Scheme 1.

### Material characterization

The crystal structure of the prepared samples was analyzed through X-ray diffraction (XRD) using a D8-ADVANCED (Bruker) instrument. The analysis was conducted at 40 kV and 30 mA with Cu Kα radiation (*λ* = 1.54178 Å) in the 2*θ* range of 5°–70° with a step interval of 0.02° and 0.25 s/step. Fourier transform infrared (FTIR) spectra were recorded in the range of 4000–400 cm^−1^ using a Bruker Vertex 70 FTIR spectrometer with KBr tablets as the medium to identify the vibrations of functional groups present in the FZC samples. Thermal stability was assessed through thermogravimetric analysis (TGA) using a TA Instruments Q500 under continuous airflow at a rate of 5 °C per minute, from 25–800 °C. Nitrogen adsorption/desorption isotherms were measured at 77 K with a Quantachrome Autosorb iQ2 analyzer to evaluate the porosity and specific surface area of the materials. The structure and surface morphology of the FZC samples were characterized *via* field emission scanning electron microscopy (FE-SEM) on a Hitachi-S4800 instrument and high-resolution transmission electron microscopy (HR-TEM) on a JEM-ARM200F (JEOL). To determine the chemical composition and electron binding energies of the materials, energy-dispersive X-ray spectroscopy (EDS) and X-ray photoelectron spectroscopy (XPS) were conducted.

### Anode preparation and electrochemical measurements

The FZC anode materials were mixed with conductive carbon (Super P) and polyvinylidene difluoride (C_2_H_2_F_2_) (10 wt%) binder mixed in *N*-methyl-2-pyrrolidone (C_4_H_7_NO) solvent with the corresponding weight ratio of 7 : 1.5 : 1.5 and the mixture stirred for 24 h. After 24 h at room temperature, the black mixture was coated on a copper (Cu) foil with a scraper and dried in a vacuum oven at 120 °C for 6 h, and the film cut with a diameter of 15 mm. Finally, the anode electrode was obtained from the FZC anode material.

Coin-type half-cells (CR-2032) were assembled in an argon-filled glovebox (MBRAUN, Germany), which consisted of a lithium foil counter electrode, the prepared anode, a polypropylene separator (Celgard 2400), and electrolyte composed of 1 M LiPF_6_ in a 1 : 1 mixture of ethylene carbonate and diethyl carbonate. The cells were cycled between 0.01 and 3 V (*vs.* Li/Li^+^) at a constant current density of 0.1 A g^−1^ using a battery cycler system (Neware, China). Cyclic voltammetry (CV) was also performed for three cycles between 0.01 and 3 V (*vs.* Li/Li^+^) on an Arbin instrument. The rate-cycling performance was evaluated at various current densities. Electrochemical impedance spectroscopy (EIS) was performed using a VSP-300 multichannel potentiostat/galvanostat (BioLogic), at frequencies in the range of 100 kHz to 100 mHz.

## Results and discussion

### Structure and morphology of α-Fe_2_O_3_–ZnO/C (FZC) nanocomposite

**Fig. 1:**
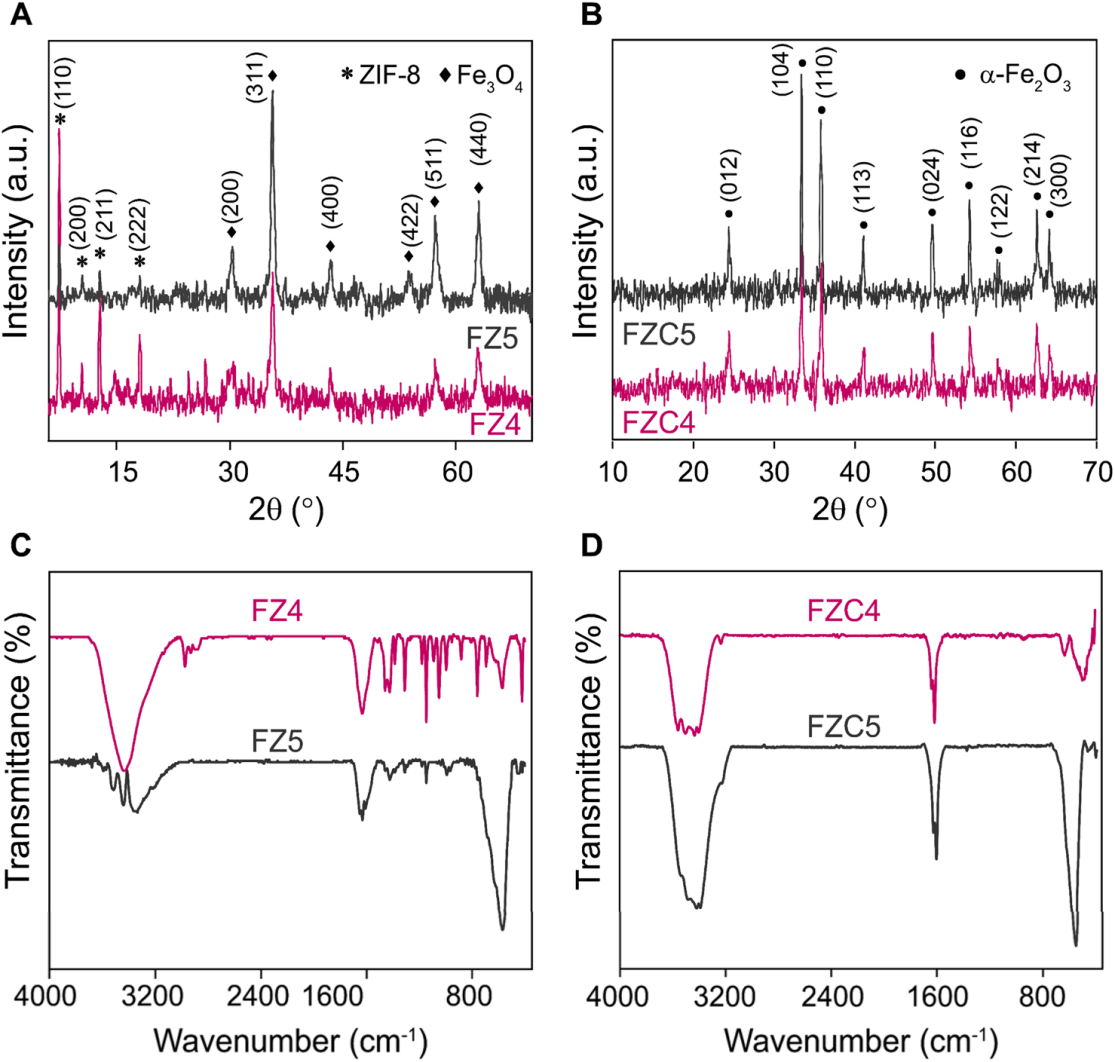
XRD patterns of (A) FZ4 and FZ5 and (B) FZC4 and FZC5. FTIR spectra of (C) FZ4 and FZ5 and (D) FZC4 and FZC5 nanocomposites.

Fig. 1A and B present the XRD patterns of the FZ and FZC nanocomposites, respectively. In Fig. 1A and S1A,† all the identified diffraction peaks correspond to the pure phases of Fe_3_O_4_, where the diffraction peaks observed at the 2*θ* values of 30.2°, 35.5°, 43.2°, 40.2°, 52.7°, and 62.9° are associated with the (200), (311), (400), (511), and (440) crystal planes, respectively, which are characteristic of a face-centered cubic (FCC) structure (JCPDS card no. 19-0629).^20,21^ Also, the broadening of the diffraction peaks related to the Fe_3_O_4_ lattice planes suggested that the nanospheres were composed of small primary nanocrystals. Meanwhile, the XRD pattern of ZIF-8 was consistent with that reported in the literature.^22,23^ The distinct diffraction peaks at 7.4°, 10.3°, 12.9°, 14.7°, 16.4°, and 18.1° correspond to the (011), (002), (112), (022), (013), and (222) planes, respectively, confirming its rhombic dodecahedral structure.^24^ However, the FZ4 and FZ5 nanocomposites exhibited distinct differences in their XRD patterns. In the FZ4 sample, the characteristic diffraction peaks of ZIF-8 were clearly observed, whereas in the case of the FZ5 sample, the diffraction peaks were predominantly attributed to Fe_3_O_4_. This result was entirely consistent with the initial prediction, which was based on the difference in the material composition ratios of the two samples. These findings verified that the phase compositions of FZ4 and FZ5 indeed consisted of Fe_3_O_4_ and ZIF-8. After heat treatment, Fig. 1B showed that all diffraction peaks for FZC4 and FZC5 are attributed to hematite (α-Fe_2_O_3_, JCPDS card no. 33-0664),^25,26^ with diffraction peaks at 2*θ* values of 24.37°, 33.35°, 35.80°, 41.02°, 49.63°, 54.22°, 57.71°, 62.58°, and 64.17°, corresponding to the crystalline planes of (012), (104), (110), (113), (024), (116), (122), (214), and (300), respectively. Additionally, the diffraction peaks of ZIF-8 vanished due to the breakdown of organic bonds under the influence of high temperature. However, in the XRD pattern of the FZC composite, the characteristic diffraction peaks of ZnO were not observed. This can be attributed to the low content of ZnO or the dispersion of ZnO within the carbon framework, resulting in weak diffraction signals that were difficult to detect.

The FTIR spectra of the FZ samples are presented in Fig. 1C. The stretching vibration at 422 cm^−1^ corresponds to the Zn–N bond in 2-methylimidazole,^27^ while the prominent peak at 580 cm^−1^ is attributed to the Fe–O stretching vibration.^28^ The bands at 760 and 690 cm^−1^ are associated with the sp^2^ C–H bending of the imidazole ring.^29^ The peak at 1590 cm^−1^ originates from the bending vibrations of adsorbed water, and the broad peak in the range of 3400–3500 cm^−1^ corresponds to the –OH group on the surface of the Fe_3_O_4_ nanoparticles.^28^ Additionally, the wide band in the range of 3200–3500 cm^−1^ corresponds to the stretching vibration of the –NH group.^29^ The FTIR spectra of FZ is consistent with the spectra of its two parent materials in Fig. S1B,† and confirming the successful synthesis of the nanocomposite, where they exhibit vibrational features consistent with both ZIF-8 and Fe_3_O_4_. Fig. 1D shows the FTIR spectra of the FZC samples, where FZC4 and FZC5 showed the disappearance of bond vibrations, leaving only Fe–O, Zn–O, C–N, C–C bonds, and O–H on the material surface, once again indicating the significant transformation of the material under the impact of high temperatures.

**Fig. 2:**
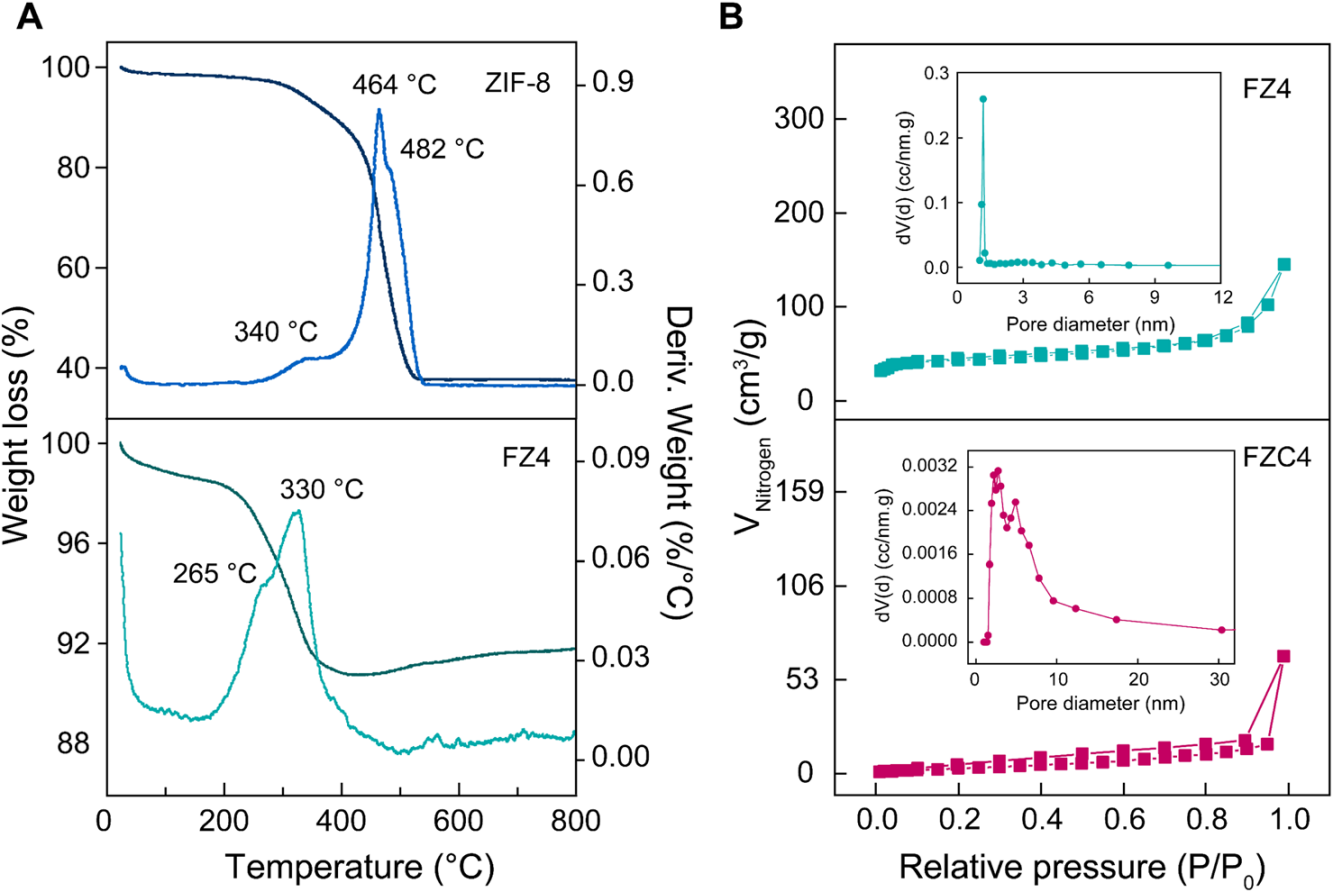
(A) TGA-DTG curve of FZ4 and ZIF-8 in an air atmosphere. (B) Nitrogen adsorption–desorption isotherms and BJH (Barrett–Joyner–Halenda theory) adsorption d*V*/d log(*D*) pore size (insert) of FZ4 and FZC4 nanocomposites.

In Fig. 2A, the blue curve represents the decomposition of the nanoporous ZIF-8, showing three distinct decomposition stages. Compared to the TGA curve of the ZIF-8 sample (blue curve), it is evident that the FZ4 composite material (green curve) exhibited higher thermal stability due to its integration with magnetic nanoparticles. Below 200 °C, the weight loss corresponded to the decomposition of guest molecules, while from 300 °C to 500 °C, the weight loss rapidly increased, and at the same time a strong peak in the DTG curve was observed, indicating the destruction of the ZIF-8 structure in an oxygen atmosphere.^30^ This process released gases such as CO_2_ and NH_3_ and left zinc oxide (ZnO), resulting in significant weight loss.^31,32^ Simultaneously, the oxidation of Fe_3_O_4_ occurred, leading to its transformation into α-Fe_2_O_3_ through the reaction 4Fe_3_O_4_ + O_2_ → 6γ-Fe_2_O_3_ → 6α-Fe_2_O_3_.^33,34^ Above 600 °C, the remaining material consisted of the stable α-Fe_2_O_3_ phase, with no significant further weight changes. Additionally, the XRD patterns of FZC-300 and FZC-500 (Fig. S2†) revealed phase transformations in the materials as the heat-treatment temperature increased, which is comparable to the data presented in earlier publications^33,34^ and the thermogravimetric results of the FZ4 composite, corresponding with the TGA-DTG analysis. The ZIF-8 nanomaterial, with its high porosity and low density, could shrink under external stimuli such as heat and chemical reactions. Specifically, high calcination temperatures could partially collapse the framework, leading to a reduction in the overall mass. MOFs in general, and ZIF-8 in particular, possess advantages such as large surface area and high pore volume, making them potential materials for LIB electrode applications when properly activated. However, these advantages can also hinder the MOF performance due to low electronic conductivity and severe irreversible lithium storage issues, together with increased side reactions such as electrolyte decomposition and SEI layer formation. Additionally, these MOFs had a low packing density and poor particle-to-particle contact, which reduced their volumetric energy density.^35^ Therefore, FZC4 and FZC5, after calcination at 700 °C, underwent partial or complete framework decomposition, resulting in a reduced BET surface area and pore volume to inter-agglomerate sintering of the nanoparticles (Fig. 2B).^36^ Simultaneously, the formation of new products such as carbon and metal oxides enhanced the electrical conductivity and minimized side reactions, thereby improving the lithium storage performance and electrode stability. According to the surface area and pore volume analysis results, it was observed that a pyrolysis time of 2.5 h at 700 °C caused the BET surface area and BJH pore volume of FZC4 to decrease compared to FZ4 from 174.58 m^2^ g^−1^ and 0.210 cm^3^ g^−1^ to 14.21 m^2^ g^−1^ and 0.107 cm^3^ g^−1^, respectively. This indicates that the high calcination temperature and prolonged calcination time led to the collapse of the ZIF-8 porous framework and reduction in surface area and pore volume. Additionally, the decomposition of organic ligand bonds and the α-Fe_2_O_3_ formed due to phase transformation during heat-treatment occupied the pores of the structures, thereby clogging them and decreased the pore size.

**Fig. 3:**
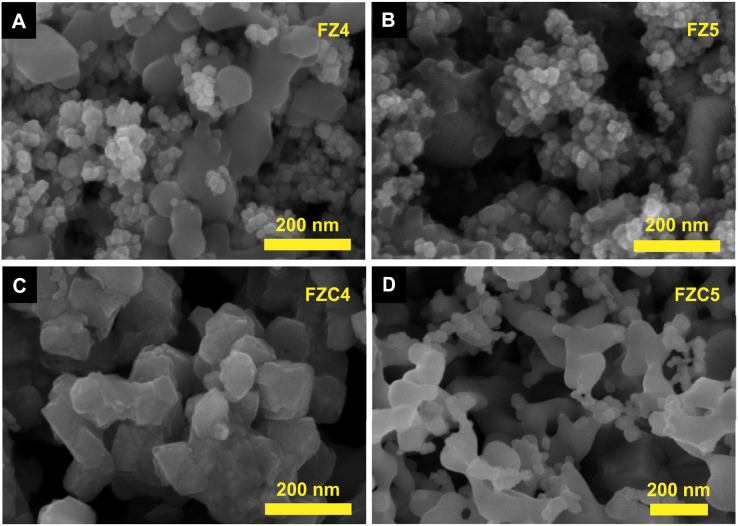
FESEM images of (A) FZ4, (B) FZ5 and (C) FZC4 and (D) FZC5 nanocomposites.

The morphology and nanostructure of the FZ and FZC nanocomposites were characterized by FESEM measurement in Fig. 3. Before the heat-treatment, FZ4 (Fig. 3A) and FZ5 (Fig. 3B) possessed Fe_3_O_4_ NPs embedded in the surface of ZIF-8, and ZIF-8 retained its uniform rhombic dodecahedral shape crystals without change due to the effect of Fe_3_O_4_ NPs. The FZ4 sample presented a relatively uniform distribution and small particle size, while the FZ5 sample showed significant agglomeration of Fe_3_O_4_ particles, forming clusters on the ZIF-8 surface. After the heat-treatment (Fig. 3C and D), the size of the nanocomposite material increased due to sintering at high temperatures, and the phase transformation of the iron oxide nanoparticles caused the rearrangement of the nanocomposite structure. The ratio difference resulted in the inability to clearly distinguish between α-Fe_2_O_3_ and the carbon skeleton component in the FZ4 sample (Fig. 3C) after the organic bonds were destroyed. Meanwhile, in the FZ5 sample (Fig. 3D), α-Fe_2_O_3_ was observed to be agglomerated and scattered on the surface of the carbon skeleton. This suggested that the α-Fe_2_O_3_ component in the FZC4 sample was better encapsulated by the carbon skeleton, providing a more stable anode material platform throughout the charge–discharge cycle, compared to the FZC5 sample, which still had excess α-Fe_2_O_3_ outside its overall structure.

**Fig. 4:**
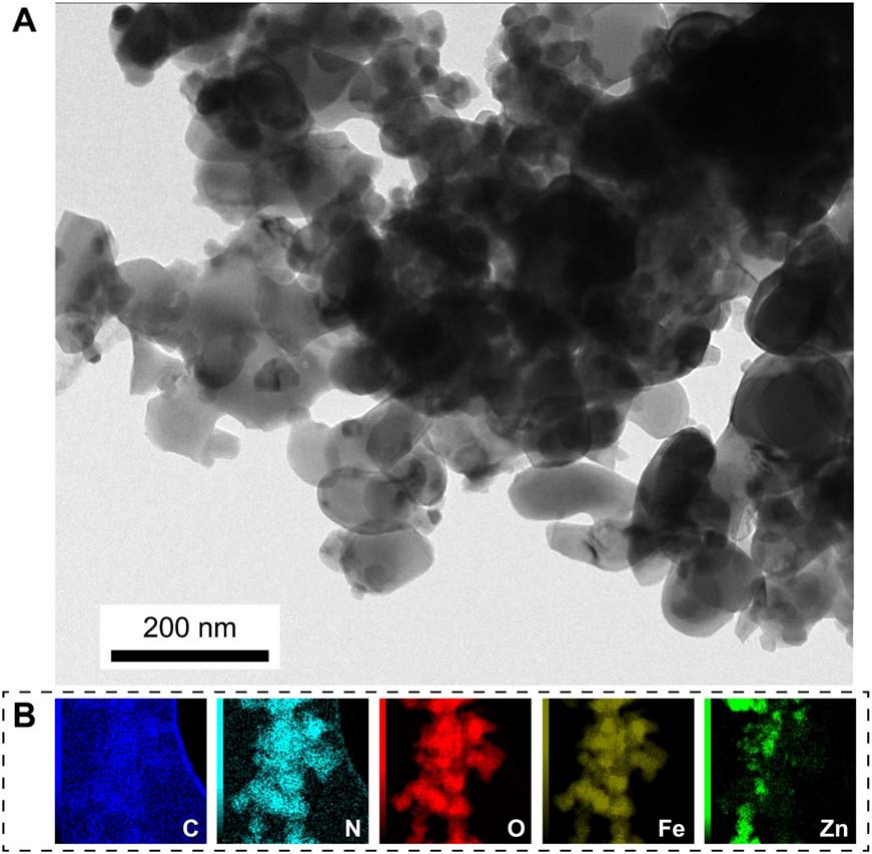
(A) TEM image and (B) EDS mapping of FZC4 nanocomposite.

Fig. 4 presents the TEM image and element mapping of the FZC4 nanocomposite. In Fig. 4A, FZC4 showed hematite nanoparticles embedded in the surface regions of ZIF-8, consistent with the observations in the FE-SEM images. It also revealed material aggregation due to sintering, leading to the formation of larger structures. The EDS elemental mapping analysis of FZC4 (Fig. 4E) revealed a relatively uniform distribution of carbon (C), oxygen (O), nitrogen (N), iron (Fe), and zinc (Zn) elements throughout its structure. Additionally, the elemental mapping showed that the distribution of Zn was relatively sparse, and less uniform compared to Fe. This result indicated that the amount of ZnO in the composite was significantly lower than that of α-Fe_2_O_3_. Furthermore, given that ZnO was formed from the thermal decomposition of ZIF-8, the ZnO particles were very small in size, leading to the absence of characteristic ZnO diffraction peaks in its XRD pattern (Fig. 1B).

**Fig. 5:**
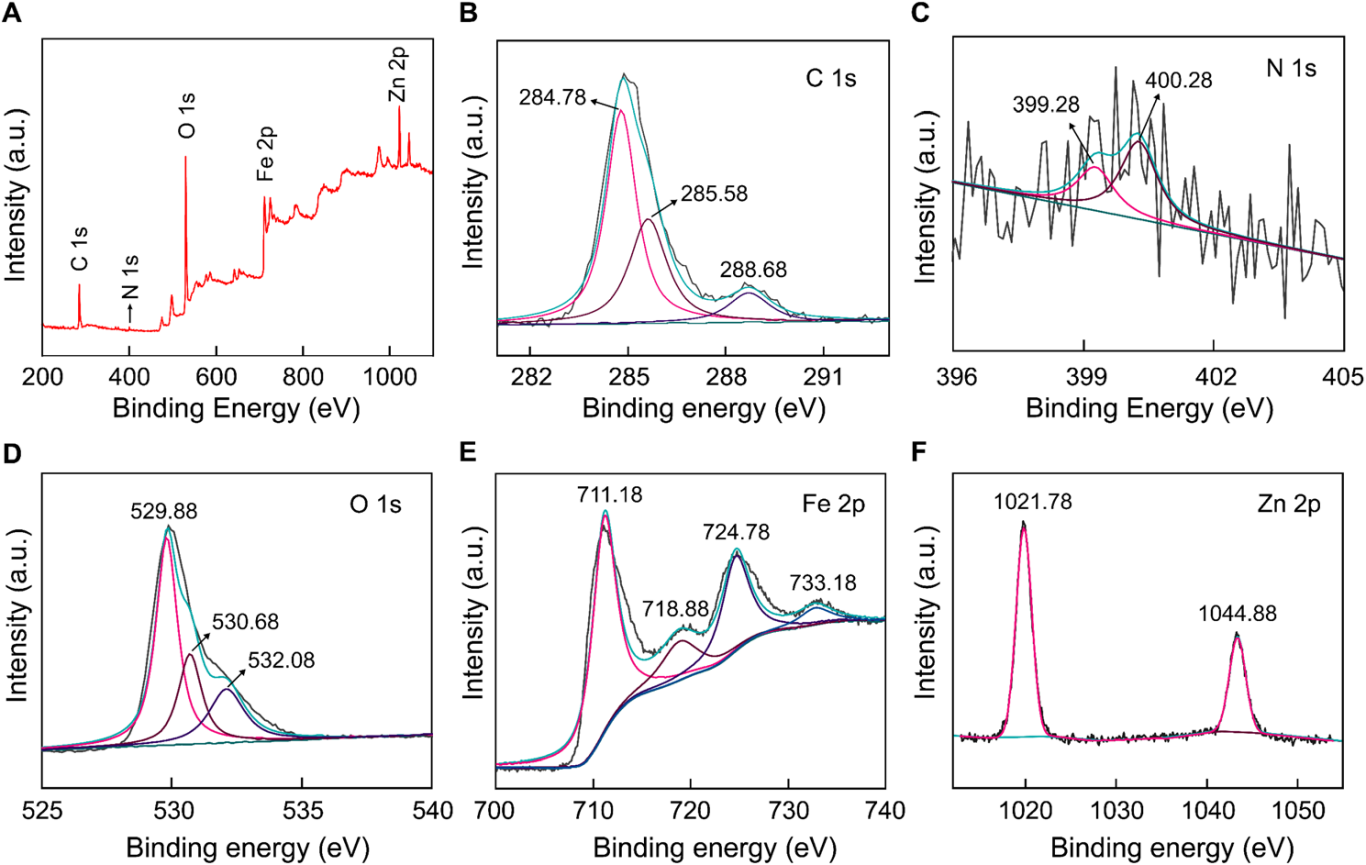
XPS spectra: (A) survey, (B) C 1s, (C) N 1s, (D) O 1s, (E) Fe 2p and (F) Zn 2p of FZC4 nanocomposite.

The valence states and elemental composition of FZC4 were more clearly observed using XPS characterization. The survey spectrum containing C 1s, O 1s, Fe 2p, and Zn 2p elements is shown in Fig. 5A. In Fig. 5B, the binding energy of C–C/C=C appeared at 284.78 eV, while C–N corresponded to the peaks at 285.58 and 288.68 eV, respectively.^37,38^ The N 1s spectrum (Fig. 5C) was deconvoluted into two distinct peaks at 399.28 and 400.28 eV, corresponding to pyridinic-N and pyrrolic-N species, respectively.^39,40^ The broad O 1s spectrum (Fig. 5D) revealed overlapping peaks at 529.88, 530.68, and 532.08 eV. The peak at 529.88 eV is characteristic of O^2−^ ions from lattice oxygen in the α-Fe_2_O_3_ matrix.^41^ The peak at 530.68 eV is attributed to oxygen vacancies in the metal oxide matrix, which are associated with oxygen defects. Anion vacancies alter the net electron charge density; this non-lattice oxygen peak is assigned to surface O^−^ ions with lower electron density. The band at 532.6 eV is related to surface hydroxyl groups (Fe–OH).^41,42^ The high-resolution Fe 2p spectrum (Fig. 5E) consisted of an Fe 2p peak split into an Fe 2p_(3/2)_ peak at 711.18 eV and Fe 2p_(1/2)_ peak at 724.78 eV. The associated satellite peaks of Fe 2p_(3/2)_ and Fe 2p_(1/2)_ were located at around 718.88 and 733.18 eV, respectively. The energy level shifted between the main peaks and the satellite peaks were located at approximately 7.7 eV for Fe 2p_(3/2)_ and 8.4 eV for Fe 2p_(1/2)_, indicating the presence of Fe^3+^ ions and conformity with the electronic state of α-Fe_2_O_3_.^42,43^ In Fig. 5F, the high-resolution Zn 2p spectrum showed two peaks located at 1021.68 and 1044.74 eV, corresponding to the Zn 2p_(3/2)_ and Zn 2p_(1/2)_ states, respectively. The spin–orbit splitting between Zn 2p_(3/2)_ and Zn 2p_(1/2)_ was 23.1 eV, indicating the existence of Zn^2+^ and the presence of ZnO.^44^ The analysis results showed that after the high-temperature annealing process, the FZC4 anode material consisted of Fe^3+^, Zn, C, O, and N, with a significant reduction in N.

### Electrochemical performance

**Fig. 6:**
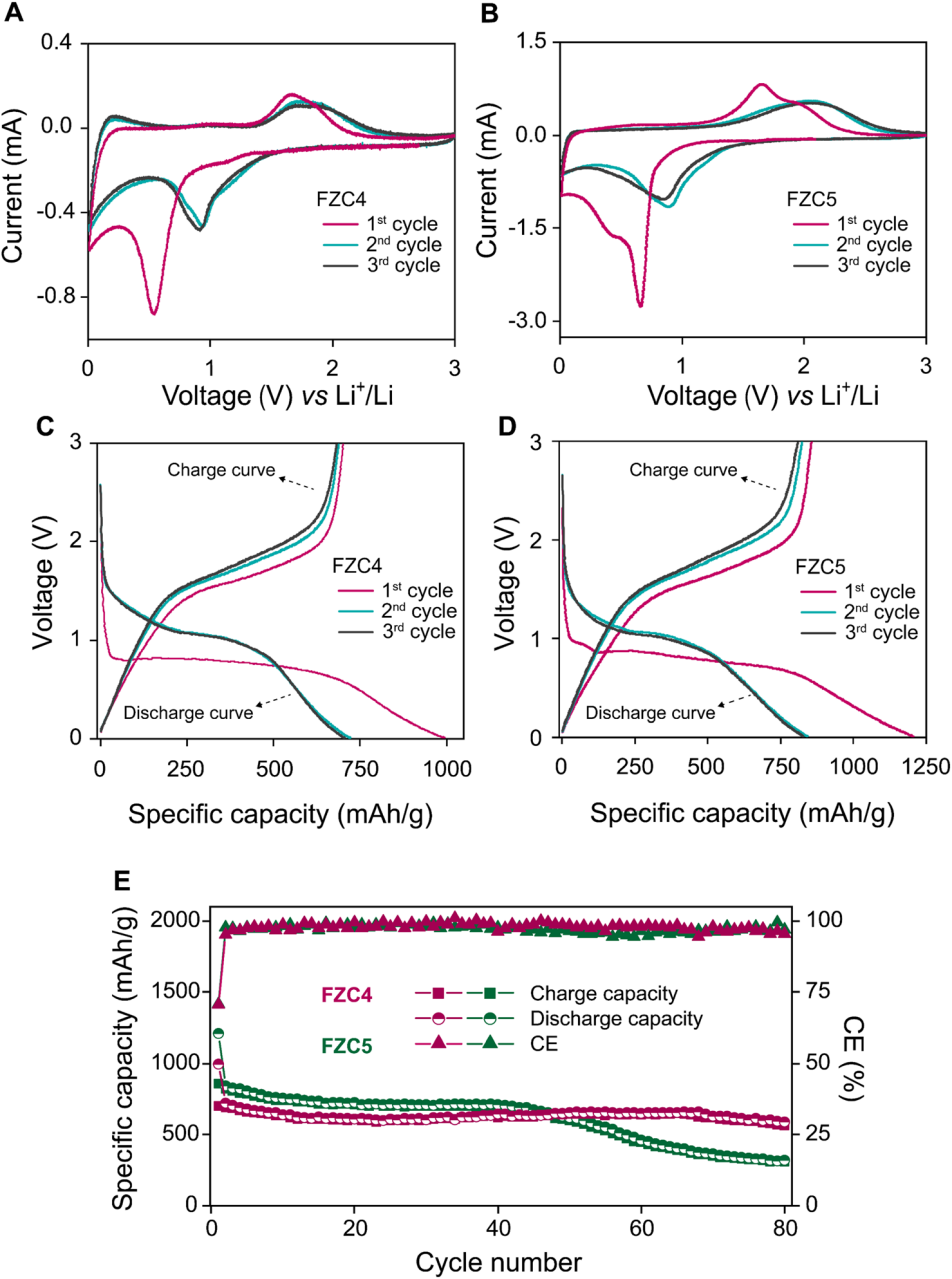
(A and B) CV curves measured at a scan rate of 0.3 mV s^−1^. (C and D) GCD tests conducted at a current density of 0.1 A g^−1^. (E) Cycling performance evaluation at a current rate of 0.1 A g^−1^ for FZC4 and FZC5.

The electrochemical properties of the FZC electrodes were first evaluated by CV measurement for the first three cycles. Fig. 6B shows the CV curves of FZC4 and FZC5 at a scan rate of 0.1 mV s^−1^ and voltage range of 0–3 V (*vs.* Li/Li^+^), respectively. In the first cathodic scan, the strong reduction peaks for FZC4 (Fig. 6A) and FZC5 (Fig. 6B) at 0.55 V and 0.65 V, respectively, were associated with the reduction of Fe^3+^ to Fe^0^ and indicated the formation of an SEI layer (Fe_2_O_3_ + 6Li^+^ + 6e^−^ → 2Fe + 3Li_2_O).^5^ In the first anodic scan, the FZC4 and FZC5 anode electrodes showed broad peaks in the range of 1.6–2.0 V, corresponding to the oxidation of Fe^0^ to Fe^3+^ and Zn^0^ to Zn^2+^.^45^ In subsequent CV cycles, the intensity of the reduction peak greatly decreased, implying that the formation of an SEI layer during the first cathodic sweep was irreversible; additionally, both the reduction and oxidation peaks shifted to higher voltages, with the reduction peak at ∼0.91 V and the oxidation peak at ∼1.72 V for FZC4, and at ∼0.89 V and ∼2.1 V for FZC5, respectively, due to the structural rearrangement of the active material and SEI layer formation from the initial electrolyte decomposition. In addition, the CV profiles of the two electrodes, FZC4 and FZC5, overlapped in the 2nd and 3rd cycles, indicating a good reversible Fe^3+^ to Fe^0^ redox reaction.^5,44,46^

Initial charge/discharge measurements of FZC4 (Fig. 6C) and FZC5 (Fig. 6D) were performed at a constant current in the potential range of 0–3 V. The first-cycle discharge/charge capacities of FZC4 were 994.4/701.1 mA h g^−1^, resulting in an ICE of ∼70.5%. The high initial capacity and degradation occurred largely irreversibly during the first cycle, primarily due to electrolyte decomposition,^47^ consumption of active lithium ions,^48^ and SEI layer formation^49^ on the anode electrode surface.^6^ Similarly, the FZC5 electrode exhibited initial discharge/charge capacities of 1206.6/855.6 mA h g^−1^ with an ICE of ∼70.9%, and its capacity degradation phenomenon was comparable to that of the FZC4 electrode. In the subsequent two cycles, a CE of ∼95% for FZC4 and ∼98% for FZC5 were achieved, indicating stable SEI layer formation on the electrode surfaces. Fig. 6E provided data on the charge and discharge cycling performance of the FZC4 and FZC5 anode electrodes in the half-cell testing, conducted at a current density of 0.1 A g^−1^. After 80 cycles, the FZC4 electrode maintained stable charge and discharge capacities of 561.2 and 587.8 mA h g^−1^, respectively, with a CE of 95.4%, indicating high structural stability and efficient electrochemical conversion. In contrast, the FZC5 electrode exhibited a significant capacity decline starting around the 40th cycle, with the charge and discharge capacities at the 100th cycle decreasing to only 318.6 and 309.5 mA h g^−1^, together with a CE of 96.9%. This disparity may reflect the inferior structural durability of the FZC5 electrode under repeated charge–discharge cycles. Furthermore, to demonstrate the impact of the heat-treatment temperature on the electrochemical performance of the FZC4 anode electrode, the FZC4-300 and FZC4-500 anode electrodes were also prepared and evaluated through the first three charge/discharge cycles and CE using the same standardized protocol (Fig. S3† and Table S1†). The results showed that both samples electrode achieved a high initial capacity due to the presence of organic bonds and elements such as N, which remained in their structure after low-temperature treatment. However, the presence of these components caused a rapid decline in capacity over the cycles, reflecting the instability of the composite material structure.

**Fig. 7:**
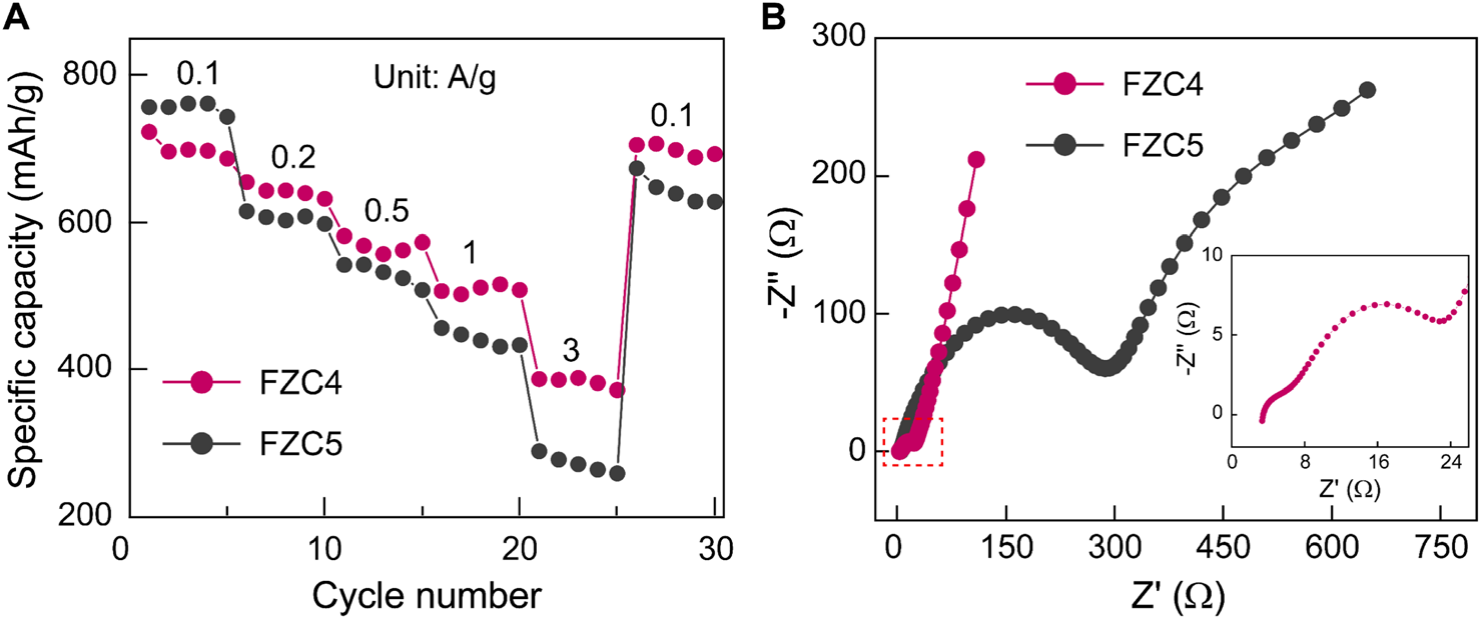
(A) Rate capability tests across varying current densities ranging from 0.1–3 A g^−1^ and (B) Nyquist plots of the FZC4 and FZC5 electrodes.

Fig. 7A illustrated the performance rate of the FZC4 and FZC5 electrodes at various current densities. The FZC4 electrode demonstrated a superior performance with charge capacities of 708.4, 642.5, 568.5, 509.0, and 382.9 mA h g^−1^ at current densities in the range of 0.1–3 A g^−1^. At high current densities, deep discharge caused particle pulverization and structural collapse, resulting in the inability to sustain a high discharge-specific capacity. However, when the current density was restored to 0.1 A g^−1^, the specific charge capacity of FZC4 recovered to 692.8 mA h g^−1^ with a capacity retention rate of 95.8%. Meanwhile, the FZC5 electrode, despite its initial charge capacity of 757.1 mA h g^−1^ at 0.1 A g^−1^, exhibited a more pronounced structural degradation at higher current densities. Its capacity progressively declined to 614.9, 542.5, 456.2, and 289.5 mA h g^−1^ at current densities in the range of 0.2–3 A g^−1^. When the current density returned to 0.1 A g^−1^, the capacity only recovered to 627.9 mA h g^−1^, with a retention rate of 82.9%. In summary, the outstanding electrochemical performance of FZC4 was attributed to its large specific surface area, which enhanced the effective contact between the electrode and electrolyte, facilitating lithium-ion access to the active sites. Additionally, the appropriate carbon content derived from the ZIF-8 framework acted as a cushioning layer, mitigating volumetric expansion and reducing the electrode impedance, thereby improving the stability and efficiency during operation.

The equivalent circuit model for the Nyquist plot is illustrated in Fig. S4,† and the EIS values are presented in Table S2.† The SEI layer resistance (R2) corresponds to the semicircle at high frequency, while the semicircle at medium frequency reflects the charge transfer resistance (*R*_3_), and the sloping line at low frequency represents the lithium-ion diffusion process (*W*). The ohmic resistance (*R*_1_) indicates the resistance of the electrolyte and other battery components.^50,51^ The EIS spectra in Fig. 7B show that FZC4 had a smaller semicircle and a higher slope compared to FZC5, confirming that FZC4 had better charge transfer capability and lower electrode/electrolyte interface resistance. Specifically, compared to FZC5, FZC4 had a lower *R*_1_ value (3.665 Ω *vs.* 5.523 Ω), indicating higher ionic conductivity in the electrolyte or improved contact between the active material and the current collector. Additionally, although the *R*_2_ value of FZC4 was slightly higher than that of FZC5 (2.676 Ω *vs.* 2.107 Ω), this suggested that the SEI layer formed on FZC4 was thicker and more stable, providing better protection for the anode, minimizing side reactions, and enhancing the long-term stability. Notably, the *R*_3_ value of FZC4 was significantly lower than that of FZC5 (8.678 Ω *vs.* 19.96 Ω), indicating a more efficient charge transfer process, which contributed to an overall improved electrochemical performance. Comparing the CE results (Fig. 6E) and rate capability (Fig. 7A), FZC4 demonstrated a superior electrochemical performance. This result also reinforced the prediction that the structure of FZC5, with a higher *R*_3_ value, tended to degrade more rapidly after multiple charge/discharge cycles. These observations underscore the critical role of the enhanced carbon content originating from ZIF-8 in stabilizing the electrode–electrolyte interfaces by mitigating the pronounced volumetric expansion of the α-Fe_2_O_3_ phase throughout cycling. Thus, the results demonstrate that the electrochemical performance of FZC4 is superior to that of FZC5.

In this study, the FZC4 anode was compared with previous research (Table S3†), which exhibited a cycling performance of 587.8 mA h g^−1^ at 0.1 A g^−1^ after 80 cycles, surpassing the *N*C-anode based on ZIF-8 (349–400 mA h g^−1^),^14,52^ most ZnO anodes (193–340 mA h g^−1^),^53–56^ and comparable to certain Fe_2_O_3_-based materials (53.42–619 mA h g^−1^).^57,58^ However, it is lower than that of ZnO nanocrystals (500 mA h g^−1^ at 0.2 A g^−1^),^59^ and significantly lower than that of thin triple shell α-Fe_2_O_3_ hollow microspheres (1702 mA h g^−1^ at 0.05 A g^−1^).^60^ Additionally, while some anodes demonstrated stable cycling up to 500–1000 cycles, the limited 80-cycle test in this work suggests a need for further long-term stability assessment. Thus, our future research will focus on optimizing the synthesis conditions with simple processes to improve the cycling stability and rate capability. Possible strategies include doping with conductive and high surface area materials (*e.g.*, MXene) to enhance the electron transport, surface modification to mitigate volume expansion, and testing at higher current densities to evaluate the rate performance. Additionally, conducting long-term cycling studies beyond 100 cycles will be essential to assess material degradation and structural integrity over extended use. These improvements can contribute to the development of more practical anode materials for lithium-ion batteries.

**Fig. 8:**
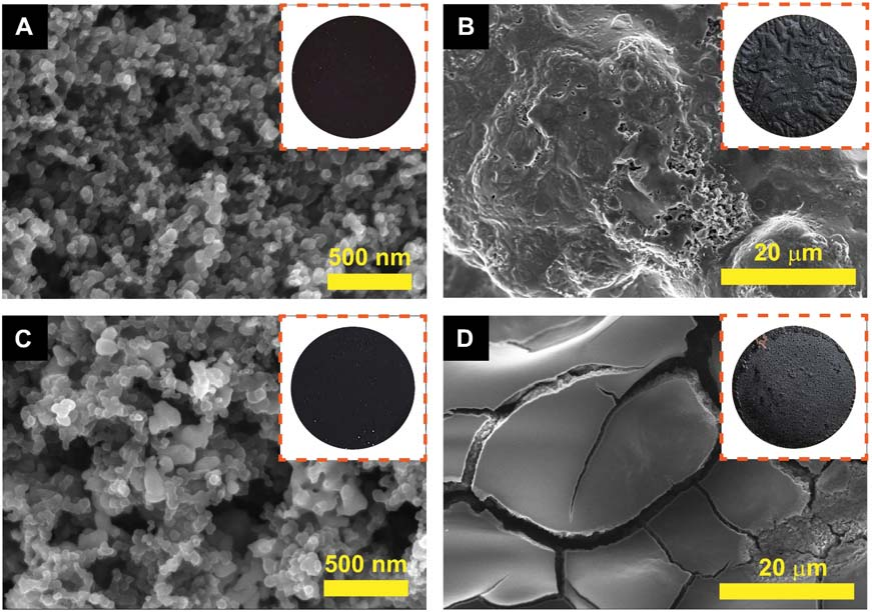
SEM images and anode electrode photographs (insets) of the FZC4 electrode (A and B) and FZC5 electrode (C and D) before and after 100 cycles at 1 A g^−1^, respectively.

To verify the structural stability of the materials, SEM analysis was performed on the electrodes by disassembling coin cells tested at 1 A g^−1^ for 100 cycles. The FZC4 and FZC5 electrodes before undergoing any electrochemical measurements are shown in Fig. 8A and C, respectively. After the charge–discharge cycles, the surfaces of the FZC4 and FZC5 electrodes were covered by a layer of material, likely the SEI layer formed during the lithiation process (Fig. 8B and D), respectively. The FZC5 electrode (Fig. 8D) was severely fragmented due to the strong volume change ofα-Fe_2_O_3_. Meanwhile, the FZC4 electrode (Fig. 8C) showed fewer surface fractures than FZC5, indicating that the ZIF-8 source covered the α-Fe_2_O_3_. However, the FZC4 electrode still exhibited some roughness and light fractures. This result is consistent with the EIS analysis, given that FESEM showed that after cycling, the SEI layer of FZC4 was thicker, providing better protection for the electrode material layer compared to that of FZC5, thereby improving the cycling ability of FZC4 over FZC5.

## Conclusions

In summary, the FZC nanocomposite material was successfully synthesized using a co-precipitation method followed by a one-step heat treatment. The ZIF-8 framework provided outstanding mechanical strength and thermal stability, which are essential for preserving the electrode integrity during repeated charge–discharge cycles. Additionally, its porous structure promoted efficient ion transport, reducing the resistance and enhancing the overall electrochemical performance. Meanwhile, α-Fe_2_O_3_ NPs, serving as the active material, exhibited excellent lithium storage capacity due to their high theoretical specific capacity. The synergistic combination of ZIF-8 and α-Fe_2_O_3_ in the FZC composite significantly improved the lithium storage efficiency, while addressing critical challenges in the charging and discharging processes of LIBs. Notably, the FZC4 anode demonstrated a reversible charge/discharge capacity of 561.2/587.8 mA h g^−1^ after 80 cycles at a current density of 0.1 A g^−1^, with low impedance and a high CE of 95.4%. These findings underscore the potential of FZC as a high-performance anode material, offering a promising avenue for advancing energy storage technologies.

## Data availability

Additional supporting data are provided in the ESI† accompanying this article.

## Author contributions

Do Thao Anh: conceptualization, data curation, formal analysis, methodology, software, writing – original draft. Nguyen Bao Tran: data curation, formal analysis, methodology. Nguyen La Ngoc Tran: conceptualization, data curation. Tran Huu Huy: methodology validation. Tran Thi Kim Chi: formal analysis, methodology. Tran Thi Huong Giang: software, validation. Tran Van Man: formal analysis, methodology. Nguyet N. T. Pham: data curation, formal analysis. Tuan Loi Nguyen: formal analysis, resources, writing – review & editing. Nhu Hoa Thi Tran: data curation, formal analysis, funding acquisition, resources, supervision, writing – original draft, review & editing.

## Conflicts of interest

The authors declare that they have no known competing financial interests or personal relationships that could have appeared to influence the work reported in this paper.

## Supplementary Material

RA-015-D5RA01206F-s001
