# Supplementary material for: ZIF-8-derived hybrid nanocomposite platform with magnetic hematite nanoparticles as enhanced anode materials for lithium storage
Source: RSC Adv. 2025 Apr 22;15(16):12746–56. doi: 10.1039/d5ra01206f (PMC12013608; doi:10.1039/d5ra01206f)
Supplement: RA-015-D5RA01206F-s001 [file RA-015-D5RA01206F-s001.pdf]

Supplementary Information (SI) for RSC Advances.
This journal is © The Royal Society of Chemistry 2025

Supplementary Information

ZIF-8-derived Hybrid Nanocomposite Platform with Magnetic Hematite Nanoparticles as Enhanced Anode Materials for Lithium Storage

Do Thao Anh[a,b,c], *Nguyen Bao Tran*[b,c], *Nguyen La Ngoc Tran*[b,c], *Tran Huu Huy*[d], *Tran Thi Kim Chi*[e], *Tran Thi Huong Giang*[e], *Van Man Tran*[b,f,g], *Nguyet N.T. Pham*[b,g], *Tuan Loi Nguyen*[h,i,*], *Nhu Hoa Thi Tran*[b,c,**]

[a]Center for Innovative Materials and Architectures (INOMAR), Ho Chi Minh City 700000, Vietnam

[b]Vietnam National University, Ho Chi Minh City 700000, Vietnam

[c]Faculty of Materials Science and Technology, University of Science, Ho Chi Minh City 700000, Vietnam

[d]Quy Nhon College of Engineering and Technology, Quy Nhon 590000, Vietnam

[e]Institute of Materials Science, Vietnam Academy of Science and Technology, Hanoi, Viet Nam

[f]Applied Physical Chemistry Laboratory (APCLAB), University of Science, Ho Chi Minh City 700000, Vietnam

[g]Department of Physical Chemistry, Faculty of Chemistry, University of Science, Ho Chi Minh City 700000, Vietnam

[h]Institute of Fundamental and Applied Sciences, Duy Tan University, Ho Chi Minh City 70000, Vietnam

[i]Faculty of Environmental and Chemical Engineering, Duy Tan University, Da Nang City 50000, Vietnam

***, ** Corresponding authors:** Tuan Loi Nguyen (Email: nguyentuanloi@duytan.edu.vn) and Nhu Hoa Thi Tran (Email: ttnhoa@hcmus.edu.vn)

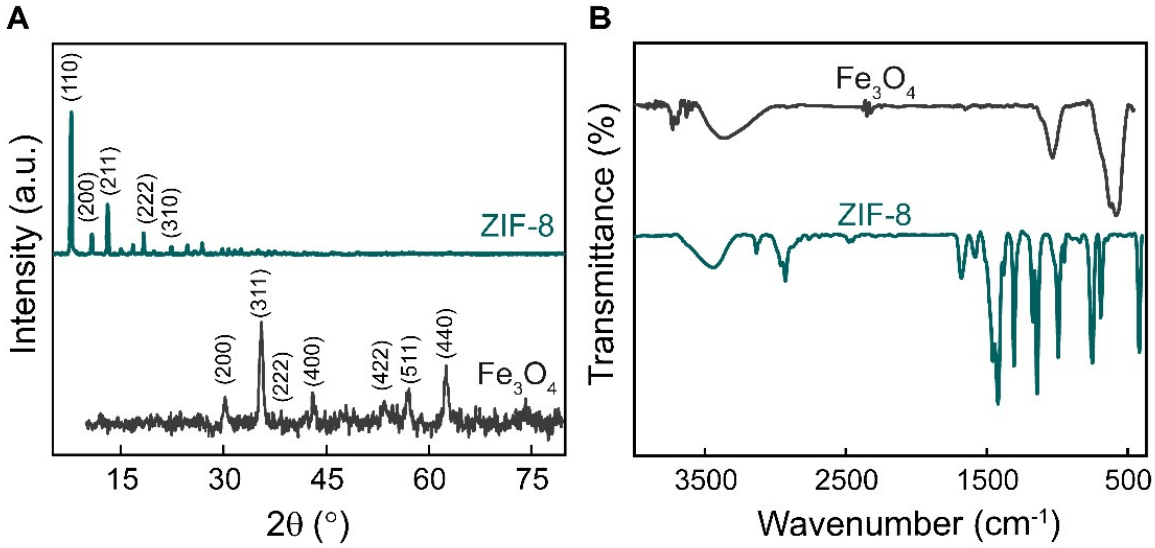

Figure S1. XRD patterns of Fe_3O_4 and ZIF-8 nanomaterial.

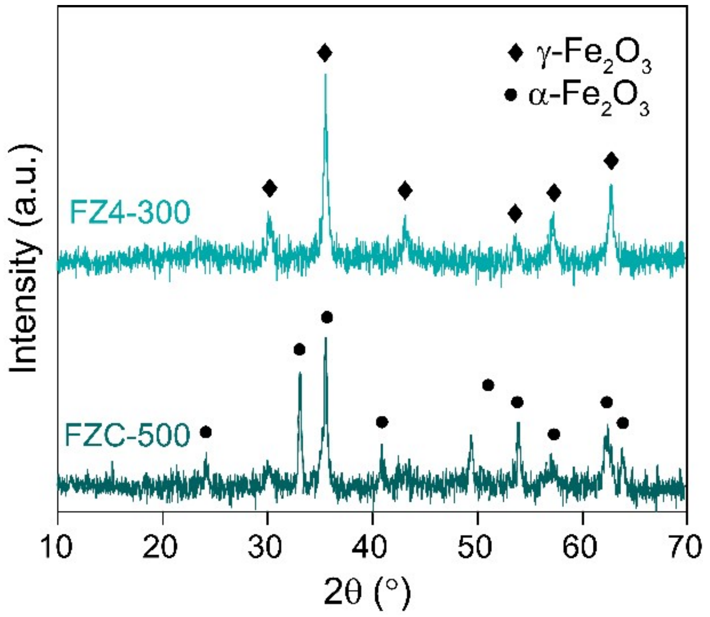

Figure S2. XRD patterns of FZC4-300 and FZC4-500 nanocomposites.

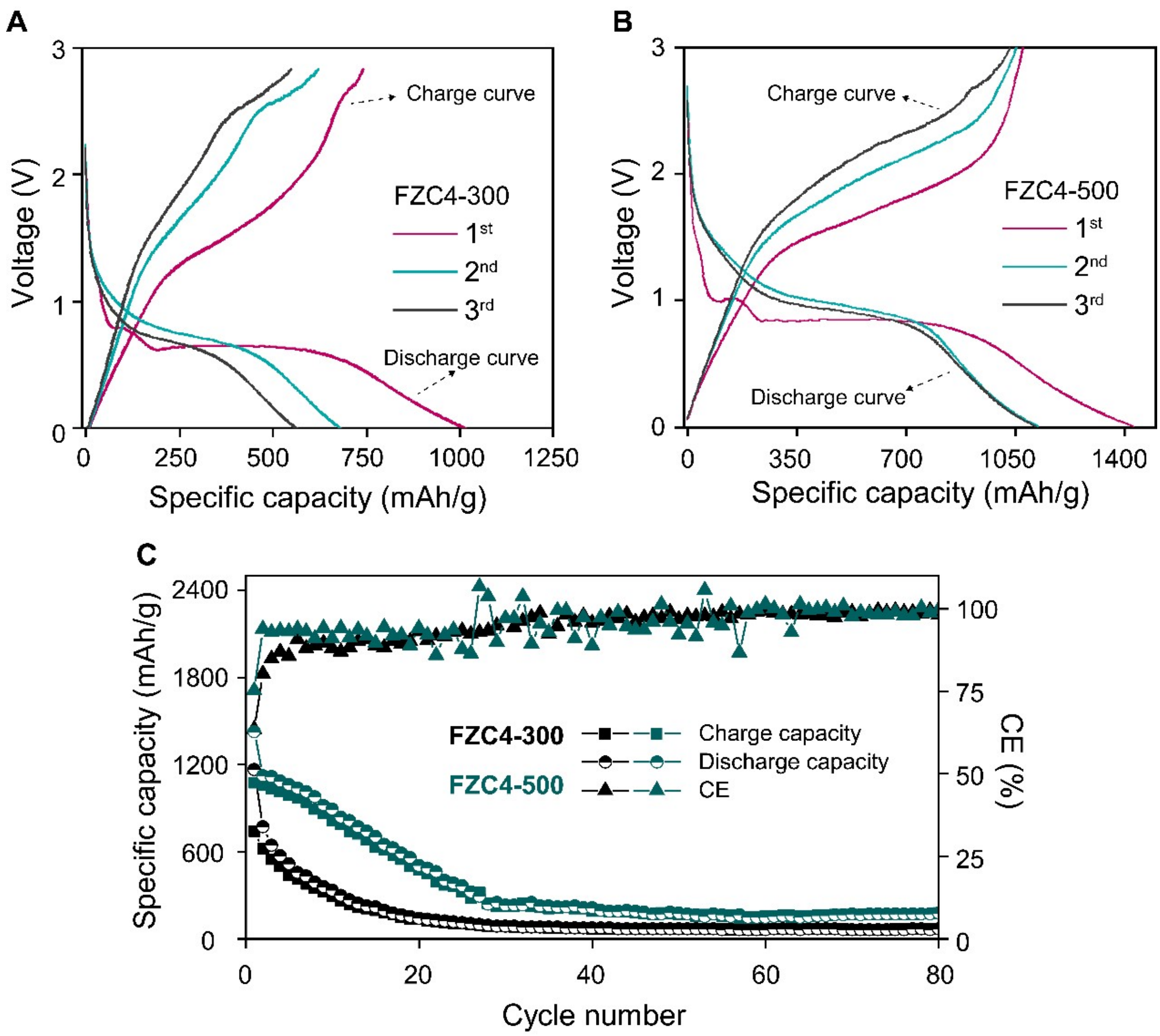

Figure S3. (A-B) GCD test and (C) cycling performances at 0.1 A/g current rate of FZC4-300 and FZC4-500 electrodes.

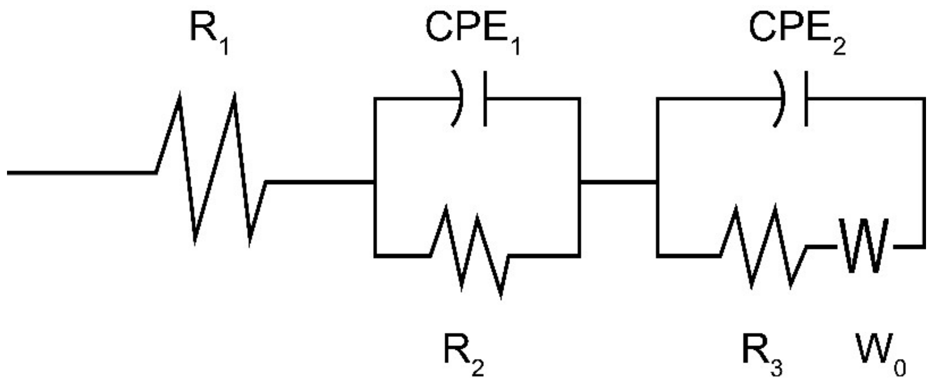

Figure S4. Equivalent circuit models were used in the analysis of anode electrodes.

Table S1. Specific capacities and CE of the initial three charge/discharge cycles for FZC4-300 and FZC4-500 anode electrodes.

Anode electrode	Cycle	Discharge capacity (mAh g^{-1})	Charge capacity (mAh g^{-1})	CE (%)
**FZC4-300**	1	1165.9	740.7	63.5
	2	773.2	620.1	80.2
	3	645.5	547.5	84.8
**FZC4-500**	1	1428.5	1075.2	75.3
	2	1123.5	1054.3	93.8
	3	1115.6	1034.0	92.7

Table S2. EIS result of FZC4 and FZC5 anode electrodes.

Anode electrode	R1 (Ω)	R2 (Ω)	R3 (Ω)
**FZC4**	3.665	2.676	8.678
**FZC5**	5.523	2.107	19.96

Table S3: Electrochemical performance comparison of ZIF-8-based, ZnO-based and Fe_2O_3-based anodes for lithium-ion batteries.

Anode material	*Cycling performance (mAh/g)*	*Current dinsity (A/g)*	*Cycle number*	*Ref.*
NC	349	0.05	50	[1]
NC-700	400	0.05	100	[2]
Bare ZnO	218	0.1	100	[3]

Bare ZnO	193	1	1000	[4]
Bare ZnO	340	1	200	[5]
ZnO/C	212	0.1	100	[6]
ZnO nanocrystal	500	0.2	100	[7]
Bare Fe_2O_3	53,42	0.2	100	[8]
Bare Fe_2O_3	619	0.5	500	[9]
Thin tripleshell a-Fe_2O_3 hollow microspheres	1702	50	50	[10]
FZC4	587.8	0.1	80	This work

References

1. Q. Li, Y. Wang, X. Gao, H. Li, Q. Tan, Z. Zhong and F. Su, *J Alloys Compd*, 2021, **872**, 159712.
2. Z. Tai, M. Shi, S. Chong, Y. Chen, C. Shu, X. Dai, Q. Tan and Y. Liu, *J Alloys Compd*, 2019, **800**, 1–7.
3. J. Park, J. B. Ju, W. Choi and S. O. Kim, *J Alloys Compd*, 2019, **773**, 960–969.
4. Q. Xu, H. Jiu, L. Zhang, W. Song, T. Gao, H. Wei, C. Wang, Y. Zhang and X. Li, *Ionics (Kiel)*, 2022, **28**, 1657–1666.
5. L. Zhang, Q. Xu, H. Jiu, W. Song, J. Yang, X. Li, H. Wei, C. Wang, X. Li and J. Zhao, *J Alloys Compd*, 2022, **915**, 165353.
6. E. Thauer, G. S. Zakharova, E. I. Andreikov, V. Adam, S. A. Wegener, J. H. Nölke, L. Singer, A. Ottmann, A. Asyuda, M. Zharnikov, D. M. Kiselkov, Q. Zhu, I. S. Puzyrev, N. V. Podval'naya and R. Klingeler, *J Mater Sci*, 2021, **56**, 13227–13242.
7. W. Zhang, L. Du, Z. Chen, J. Hong and L. Yue, *J Nanomater*, 2016, **2016**, 8056302.
8. X. Liu, K. Xiong, H. Yuan and J. Zhao, *Ionics (Kiel)*, 2024, **30**, 1373–1381.
9. L. Hu, J. Huang, Z. Yang, J. Li, P. Wang, L. Wang and P. Sun, *Solid State Ion*, 2022, **383**, 115981.
10. S. Xu, C. M. Hessel, H. Ren, R. Yu, Q. Jin, M. Yang, H. Zhao and D. Wang, *Energy Environ Sci*, 2014, **7**, 632–637.
